# Supplementary material for: Loss of the Extracellular Matrix Protein DIG-1 Causes Glial Fragmentation, Dendrite Breakage, and Dendrite Extension Defects
Source: J Dev Biol. 2021 Oct 7;9(4):42. doi: 10.3390/jdb9040042 (PMC8544517; doi:10.3390/jdb9040042)
Supplement: Supplementary file 1 [file jdb-09-00042-s001.zip › jdb-1359532-supplementary.pdf]

Supplemental Material

Loss of the extracellular matrix protein DIG-1 causes glial fragmentation, dendrite breakage, and dendrite extension defects

Megan K. Chong, Elizabeth Cebul, Karolina Mizeracka, and Maxwell G. Heiman

Supplemental Figure S1

Tables S1-S3

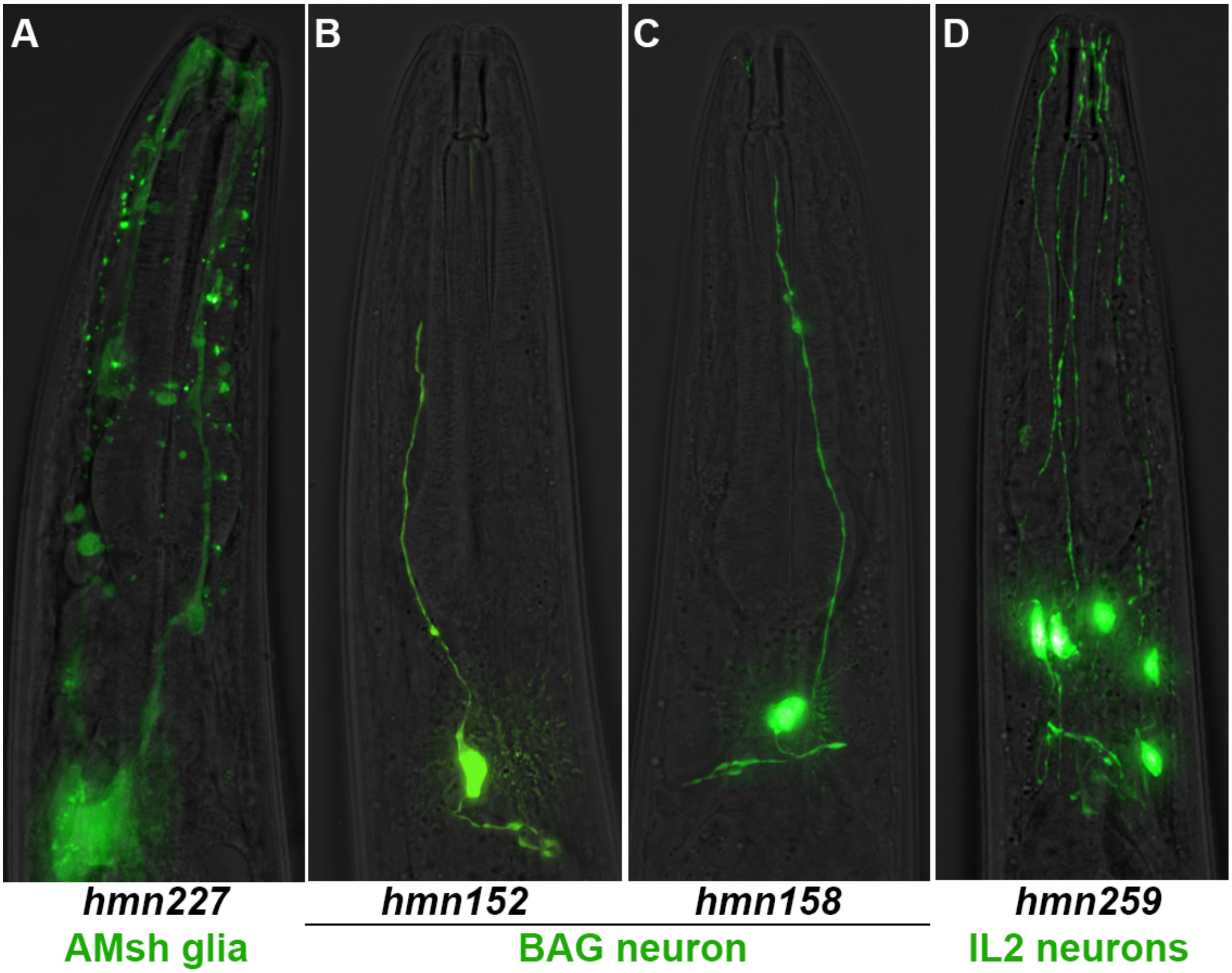

Supplemental Figure S1. Isolation of mutants with altered glial or neuronal morphology.

Adult animals exhibiting phenotypes identified in the genetic screen: (A) amphid sheath glial fragmentation in *hmn227*; (B, C) BAG dendrite length defects in (B) *hmn152* and (C) *hmn158;* *(*D) IL2 dendrite fasciculation defects in *hmn259*.

Table S1. Strains used in this study

Strain	Genotype	Figure(s)
CHB1652	*dig-1(hmn227)* III*; hmnIs13*	1
CHB1166	*dig-1(hmn152)* III*; oyIs82* X	1
CHB2250	*dig-1(hmn158)* III*; oyIs82* X	1
CHB3008	*dig-1(hmn259) myIs13* III	1
CHB1549	*hmnIs13*	2
CHB4342	*dig-1(n1321)* III*; hmnIs13*	2
PY8503	*oyIs82* X	3
CHB4343	*dig-1(n1321)* III*; oyIs82* X	3
PT2762	*myIs14* IV	4, 5
CHB2940	*dig-1(n1321)* III*; myIs14* IV	4, 5
CHB2937	*myIs14* IV; *dyf-7(ns119)* X	5
CHB2944	*dig-1(n1321)* III*; myIs14* IV; *dyf-7(ns119)* X	5

Table S2. Transgenes used in this study

Transgene	Description	Reference
hmnIs13	*F16F9.3*pro:mCherry, *grl-2*pro:YFP, *gcy-8*pro:CFP	Mizeracka et al., bioRxiv 2019
oyIs82	*flp-17*pro:GFP, *unc-122*pro:dsRed	Gift of Astrid Cornils and Piali Sengupta
myIs13	*klp-6*pro:GFP	Schroeder et al., Curr. Biol. 2013
myIs14	*klp-6*pro:GFP	Schroeder et al., Curr. Biol. 2013

Table S3. Alleles generated in this study

Allele	Sequence
dig-1(hmn152)	CTGATGGT[C>T]AGATTCTT
dig-1(hmn158)	ATGGACAG[g>a]tacgatta
dig-1(hmn227)	ATGGACAG[g>a]tacgatta
dig-1(hmn259)	GGTTCAC[C>T]CAATAACA

Substitutions are bracketed. Uppercase corresponds to predicted exons; lowercase to predicted introns. *hmn158* and *hmn227* were isolated from independent screens but cause an identical nucleotide change.
